# Childhood colostomies: patterns, indications and outcomes in a Nigerian University Teaching Hospital

**DOI:** 10.4314/ahs.v22i4.25

**Published:** 2022-12

**Authors:** Uchechukwu Obiora Ezomike, Ijeoma Esther Nwachukwu, Emmanuel Ifeanyi Nwangwu, Isaac Sunday Chukwu, Samson Chukwuemeka Aliozor, Elochukwu Perpetua Nwankwo, Sebastian Okwuchukwu Ekenze

**Affiliations:** Sub-Department of Pediatric Surgery University of Nigeria Teaching Hospital Ituku/Ozalla Enugu, Nigeria

**Keywords:** Childhood colostomies, oatterns, indications, outcomes

## Abstract

**Background:**

Most childhood colostomies are done for decompression or diversion in gastrointestinal tract congenital anomalies. Colostomy may be sited in the transverse or sigmoid colon as loop or defunctioning (divided) colostomies. Current pattern seems towards construction of more sigmoid and defunctioning colostomies.

**Aims:**

To evaluate the patterns, indications and outcomes of childhood colostomies. Patients and Methods: Retrospective chart review of all colostomies performed in children below 15 years from September 2010 to August 2020.

**Results:**

There were 104 colostomies (55males; 49females; 65 sigmoid; 39 transverse colostomies; 3 loop; 101 defunctioning colostomies. Anorecatal Malformation (ARM)was indication in 32 males and 41 females; age range 2 days to 13 years. Hirschsprung's Disease (HD) was indication in 18 males and 4 females; age range 6 weeks to 15 years.

In HD there were three loop colostomies (3/22) in transverse colon and 19 defunctioning colostomies (8 sigmoid, 11 transverse) while in ARM all 73 were defunctioning colostomies(P=0.01)

In HD there were 14/22 transverse colostomies and 8/22 sigmoid colostomies while ARM had 24/73 transverse and 49/73 sigmoid colostomies (P =0.013)

In HD 91% colostomies were done beyond infancy while in ARM 93% were before one year(P<0.0001). Mortalities were noted in 1.9% patients.

**Conclusion:**

Commonest indication for colostomy is ARM. There are more defunctioning than loop colostomies, and more sigmoid than transverse colostomies. of most colostomies in ARM were during infancy while mostly beyond infancy in HD.

## Introduction

In the pediatric age group, indications for colostomies vary and may be for congenital or acquired anomalies, though congenital conditions seem more likely^1^,^2^,^3^ They are constructed where there is need to decompress the lower gastrointestinal tract or divert stool from getting to a distal site, like in fistulous communication between bowel and the urinary tract, to avoid fecal contamination. Colostomy may be sited in the transverse or sigmoid colon and may be contracted as loop or defunctioning (divided) colostomy. The congenital indications are mainly anorectal malformation (ARM) and Hirschsprung's disease (HD), but the relative contribution of each as indication varies among various publications with HD more in some^1^,^4^,^5^ and ARM more in others^2^,^3^,^6^,^7^. Incidence of colostomy for HD is dropping in some countries due to early presentation and performance of more single stage transanal pull-through procedures^8^,^9^,^10^ but initial colostomy is still high in our environment^11^ and other Low and Middle Income Countries^12^,^13^,^14^ where late presentation is still rife. In the past there used to be many transverse^1^,^2^ and loop colostomies^1^,^2^ but the current trend seems to be more towards construction of defunctioning and sigmoid colostomies^3^,^14^,^15^,^16^. We therefore set out to study the trend in our hospital comparing present study with a previous study from our Centre and with other available literature.

## Patients and methods

This is a retrospective review of all colostomies performed in our hospital on children below 15 years from I^st^ September 2010 to 31^st^ August 2020. Information on indications for colostomy, age at colostomy, anatomical site of colostomy, methods of construction and complications were retrieved from patients' folders and theatre records. Data entry and analysis were done using Statistical Package for Social Sciences (IBM SPSS) version 20 (IBM Co., Armonk, NY, USA). The results were presented as means, ranges, percentages, tables. Ethical clearance for the study was obtained from the Health Research and ethics Committee of our institution.

## Results

There were one hundred and four (104) colostomies carried out in fifty-five (55) males and forty-nine (49) females aged 2days to 15years with sixty-five (65/104,62.5%) sigmoid and thirty-nine (39/104,37.5%) transverse colostomies; three loop (3/104,2.9%) and one hundred and one (101/104,97.1%) defunctioning colostomies. (Table 1)

**Table 1:** Age and Sex distribution of Indications for colostomy in children

Age	Anorectal malformation		Hirschsprung's Disease		Others		Total
	Male	Female	Male	Female	Male	Female	
**<1month**	**31**	10	-	-	1	-	42
**>1month**–**12months**	1	**26**	2	-	2	-	31
**>12months**–**5years**	-	4	**12**	-		1	17
**>5years**–**10years**	-	-	3	3	2	3	11
**>10years to** **15years**	-	1	1	1	-	-	3
**Total**	32	41	18	4	5	4	104

The indication for colostomy was ARM in seventy-three patients (73/104,70.2%) (32 males, 41 females) (table 2). Ages ranged from 2days to 13years (modal age is 3 days, median age 7 days, interquartile range 4days -6months). There were no loop colostomies in ARM; 49 were sigmoid defunctioning colostomies while 24 were transverse defunctioning colostomies. In 93% (68/73) of the ARM, colostomies were done before 1year of age. In 7% (5/73) of ARM, however, colostomy was done beyond one year. All the colostomies done beyond one year were all females with one rectoperineal fistula and four recto vestibular fistulas.

**Table 2:** Showing differences between colostomy in HD and Anorectal Malformation

	Hirschsprung's disease (N=22,18males, 4 females)	Anorectal malfor mation (N=73, 32 males, 41 females)	P-value
**Median Age**	4.95years	7days	
**Colostomy type**			
**Defunctioning colostomy**	19	73	0.01
**Loop colostomy**	3	0	
**Anatomical site of colostomy**			
**Transverse colostomy**	14	24	0.013
**Sigmoid colostomy**	8	49	
**Number of colostomies done** **beyond 1year of age**	20/22(91%)	5/73(6.8%)	<0.0001
**Mortality**	0	2	

Hirschsprung's Disease (HD) was indication for colostomy in twenty-two patients (22/104,21.2%) (18 males and 4 females) (table 2) with age range from 6weeks to 15years (Mean 4.6 years, median 4.95 years, mode 5 years, interquartile range 2 years to 8 years).

In HD there w ere three loop colostomies (3/22) in the transverse colon and 19 defunctioning colostomies (8 sigmoid, 11 transverse) while in ARM all 73 were defunctioning colostomies(P=0.01)

In HD there were 14/22 transverse colostomies and 8/22 sigmoid colostomies while in ARM there were 24/73 transverse and 49/73 sigmoid colostomies (P =0.013)

In HD 20/22 colostomies were done beyond one year of age, while in ARM 68/73 colostomies were done before one year(P<0.0001).

In the first half of the 10year period of study, more senior registrars constructed colostomies than consultants while in the second half more consultants than senior registrars created the colostomies () p<0.0001). Also, the overall mean age at colostomy(15months) in the second half of the period was significantly lower than mean of 24months in the first half(p=0.036). The noted complications were also more in the first half than in the second half. There was no significant difference in the type of colostomy (p=1.00), anatomic site of colostomy (p=0.57), indication for colostomy (p=1.00), sex distribution (p=0.44), incidence of colostomy revisions (p=0.57) between the first and second halves of the study period. Other less common indications in 5 males and 4 females were 6 cases of trauma comprising Traumatic anal sphincter injury (1), Rectal impalement injury (1), Penetrating deep perineal injury (1), Traumatic posterior urethral stricture (1), transabdominal Penetrating sigmoid injury (2)], complicated ileocolic intussusceptions (2), sigmoid perforation in congenital anterior abdominal wall defect (1). All except one were sigmoid defunctioning colostomies.

Forty-four patients (42%, 24 sigmoid and 20 transverse) had 63 complications. (Table 3)

**Table 3:** Observed Complications of Colostomy

Peristomal skin excoriation	**25**
Superficial skin bridge breakdown	12
Stomal prolapse	9
Stomal retraction	4
Stomal stenosis	3
Burst abdomen	3
Stomal bleeding	2
Parastomal hernia	2
Surgical site infection	2
Stomal necrosis	1
**Total**	63

Most common was peristomal skin excoriation which resolved over time with application of various salves and proper skin care.

There were six reoperations following the colostomies. The re-operations were all in sigmoid defunctioning colostomies and indications included stomal retraction (1), massive stomal prolapse (1), Complete breakdown of the skin bridge in the early postoperative period (burst abdomen) (3), obstructing parastomal hernia (1)

Colostomy-related mortality occurred in two neonates with ARM, one male and one female (2/104, Mortality rate =1.9%). Both had sigmoid defunctioning colostomies. One had sepsis with burst abdomen and small bowel perforation while the other had sepsis with stomal necrosis.

## Discussion

The commonest indication for colostomy in this study is anorectal malformation. This seems to be the current trend as is seen in some other studies^2^,^3^,^6^,^16^. In some earlier publications^1^,^4^,^5^, however, more colostomies for HD than ARM were noted. The second most common indication in this study is HD as was also in some other reports^2^,^3^,^16^ while 3rd most common indications are various forms of trauma as also seen in some works^2^,^17^.

In the current study median age at colostomy in ARM is 7days. In an earlier study Ekenze et al^1^ had a mean age at colostomy of 15days in ARM. This implies a reduction in age at surgery for ARM but this is still higher than a median of 4 days recorded by Lukong et al^15^ in Northern Nigeria and comparable to median of 6days by Almossalam et al^18^ in Saudi Arabia. Our study found that a few cases of ARM had colostomy beyond 1year of age unlike in Ekenze et al^1^ where all ARM had colostomy before the age of 3months and Nour et al^5^ where all colostomies were done below the age of one year. The colostomies done beyond one year of age for ARM in this study were all in females with rectoperineal and rectovestibular fistulae. Muzira et al^6^ also reported many colostomies in ARM beyond one year of age.

This study shows that median age for colostomy in HD is 4.95 years. This is similar to the mean age at colostomy of 4.6 years in an earlier study from same centre^1^ but higher than median age of 2 years noted by Mabula et al^12^ in Tanzania. In this study, majority of HD had colostomy after 1year of age as also recorded in some other studies^1^,^6^ while in another study^5^ only 4.7% of HD patients had colostomy after 1 year. Late presentation in HD is still a challenge^11^ and this may be because most times decision to investigate and diagnose HD is delayed due to the common belief that some degree of constipation is normal in infants. Furthermore, there is delayed referral for pediatric surgical care even when patients present early to a health facility^11^.

In this study male to female ratio is 0.8: 1 in ARM and is 4.5:1 in HD while overall male to female ratio is 1.1: 1. This shows that there are more females in this report than many others with much higher male: female ratios^1^,^19^,^20^. This may be explained by the greater number of females with ARM in this study. In some other studies more males than females required colostomies for ARM^1^,^18^.

Anatomic site of colostomy in this study was more in the sigmoid colon as opposed to an earlier study from same centre^1^ and other centres^2^,^17^,^20^ where colostomies were mainly constructed in the transverse colon. This finding is in consonance with some other publications where there were also more sigmoid colostomies^3^,^6^,^21^. Although, in the absence of facilities for frozen section Ekenze et al^1^ encouraged construction of colostomy in transverse colon to ensure that the colostomy is constructed at a point adequately proximal to a ganglionic bowel, the current trend seems to be more towards construction of more sigmoid than transverse colostomies in HD. Furthermore, in the absence of frozen section primary trans anal pull through surgery may be fraught with a high incidence of constipation due to residual a ganglionic or hypo ganglionated pulled-through colon. Hence with late presentation, usually associated with dilated colon, sigmoid colostomy using the apparently ganglionated bowel for stoma creation is done coupled with biopsy and histology to confirm presence of ganglion cells at the stoma site and then later pull through of stoma is done. This pattern of care has also encouraged more of sigmoid colostomy in short segment HD in our hospital. Other studies have also discouraged construction of transverse colostomy^3^,^7^. This is in tandem with other more recent studies where there seems to be a general trend towards more sigmoid than transverse colostomy^3^,^6^ in most ARM and HD. In cloacal anomaly, however, transverse colostomy may still be preferable to preserve the sigmoid colon in case there is need for substitution vaginoplasty using sigmoid colon.

In this study, loop colostomy was done only in 2.9% of patients. When compared with an earlier publication from the same centre^1^ where about 49.50% of all their colostomies were of the loop type, there has been a major drop in the rate of performing loop colostomies in our centre. This may be related to higher incidence of complications in loop versus defunctioning colostomies^1^,^5^,^22^,^23^ necessitating a change in practice by the surgeons. All the loop colostomies in this study were in the transverse colon and for HD and none was for ARM. In an earlier publication from the same centre^1^, some transverse loop colostomies were done in ARM and few sigmoid loop colostomies were also done. Some authors believe that all ARM with suspected fistula to urinary tract should have defunctioning colostomies and not loop colostomies to prevent continuous fecal urinary tract contamination^1^,^24^. Defunctioning colostomy is the main type of colostomy in this study and colostomies in ARM are all of the defunctioning type. Defunctioning colostomy was also the preferred type in ARM in a metanalysis by Youseff et al^25^. In a review by Levitt^24^, preferred colostomy in ARM was the defunctioning type and loop colostomy was discouraged as it does not completely divert stool and this may lead to fecal contamination of the urinary tract. Chirdan et al^21^ also had more sigmoid defunctioning colostomies in ARM.

Complications associated with colostomy negatively affect the quality of life of those on colostomy and efforts should be made to reduce them. Transverse loop colostomies has more complications^1^,^2^,^5^,^7^ and this may explain the drop in usage in our study.

Peristomal skin excoriation was the commonest complication in this study. It was also the most common in some other studies^1^,^2^,^7^,^22^. Peristomal skin excoriation is mainly caused by prolonged stool contact with peristomal skin. This occurs especially when there is lack of appropriate stoma appliances, and is made worse when there is colostomy diarrhea. Despite being the most common complication, incidence may be underreported as retrospective analysis may underestimate minor complications like skin excoriation^5^.

The second most common complication in this study is superficial skin bridge breakdown which mostly healed with adequate peristomal skin care.

Prolapse is the third most common complication in this study as opposed to some other publications^1^,^2^,^7^ where it is second most common complication. The lower rate of prolapse as compared with other studies^1^,^2^,^7^ may be due to lower rates of transverse loop colostomies in this study. Reoperations were done in 5.8% of patients and all were in sigmoid defunctioning colostomies. This is lower than the reoperations rates of 8.5% found in Ekenze et al1 and 7.5% found by Dode et al^20^ but slightly higher than 5.5%2 noted in another study by Ciğdem et al. This finding of reoperations being more in sigmoid than transverse colostomies may be related to the fact that sigmoid defunctioning colostomy was the most commonly performed colostomy in this study and loop colostomy which is usually associated with most complications^1^,^2^,^5^,^22^ is quite low in this study.

Mortality directly related to colostomy was seen in 1.9% of the patients and is comparable to rate of 1.6% noted in an earlier study from same centre1 and 1.5% by Dode et al^20^ but lower than mortality rate of 2.7% recorded by Ciğdem et al^2^. All the noted mortalities in this study were in neonates. Most mortalities in another study^2^ were also in neonates.

The limitations of this study is the fact that it is a retrospective single center study.

## Conclusion

Colostomy in children is done mainly for congenital anomalies, especially anorectal malformation (ARM). Colostomy in general is constructed more in males than females but in ARM more colostomies were constructed in females than males. There are more defunctioning than loop colostomies as well as more sigmoid and less transverse colostomies. In ARM there were no loop colostomies. Most colostomies in ARM are before one year while most colostomies for HD are after one year.

## Recommendations

We recommend a prospective study of indications and outcomes of childhood colostomies in our environment.

## References

[1] Ekenze SO, Agugua-Obianyo NE, Amah CC (2007). Colostomy for large bowel anomalies in children: a case-controlled study. Int J Surg.

[2] Ciğdem MK, Onen A, Duran H, Oztürk H, Otçu S (2006). The mechanical complications of colostomy in infants and children: analysis of 473 cases of a single center. Pediatr Surg Int.

[3] Massenga A, Chibwae A, Nuri AA, Bugimbi M, Munisi YK, Mfinanga R (2019). Indications for and complications of intestinal stomas in the children and adults at a tertiary care hospital in a resource-limited setting: a Tanzanian experience. BMC Gastroenterol.

[4] Ameh EA, Mshelbwala PM, Sabiu L, Chirdan LB (2006). Colostomy in children--an evaluation of acceptance among mothers and caregivers in a developing country. S Afr J Surg.

[5] Nour S, Beck J, Stringer MD (1996). Colostomy complications in infants and children. Ann R Coll Surg Engl.

[6] Muzira A, Kakembo N, Kisa P, Langer M, Sekabira J, Ozgediz D (2018). The socioeconomic impact of a pediatric ostomy in Uganda: a pilot study. Pediatr Surg Int.

[7] Soomro BA, Solangi RA, Siddiqui MA (2010). Colostomy in Children: Indications and Complications. Pak J Med Sci.

[8] Giuliani S, Honeyford K, Chang CY, Bottle A, Aylin P (2020). Outcomes of Primary versus Multiple-Staged Repair in Hirschsprung's Disease in England. Eur J Pediatr Surg.

[9] Nouira F, Ben Ahmed Y, Sarrai N, Ghorbel S, Jlidi S, Khemakhem R (2012). Surgical management of recto-sigmoid Hirschsprung's disease. Acta Chir Belg.

[10] Sulkowski JP, Cooper JN, Congeni A, Pearson EG, Nwomeh BC, Doolin EJ (2014). J Pediatr Surg.

[11] Ekenze SO, Ngaikedi C, Obasi AA (2011). Problems and outcome of Hirschsprung's disease presenting after 1 year of age in a developing country. World J Surg.

[12] Mabula JB, Kayange NM, Manyama M, Chandika AB, Rambau PF, Chalya PL (2014). Hirschsprung's disease in children: a five-year experience at a university teaching hospital in north-western Tanzania. BMC Res Notes.

[13] Sharma S, Gupta DK (2012). Hirschsprung's disease presenting beyond infancy: surgical options and postoperative outcome. Pediatr Surg Int.

[14] Bandré E, Kaboré RA, Ouedraogo I, Soré O, Tapsoba T, Bambara C (2010). Hirschsprung's disease: management problem in a developing country. Afr J Paediatr Surg.

[15] Lukong CS, Ameh EA, Mshelbwala PM, Jabo BA, Gomna A, Akiniyi OT (2011). Management of anorectal malformation: Changing trend over two decades in Zaria, Nigeria. Afr J Paediatr Surg.

[16] Anyanwu LJ, Mohammad A, Oyebanji T (2013). A descriptive study of commonly used postoperative approaches to pediatric stoma care in a developing country. Ostomy Wound Manage.

[17] Adotey JM (1998). Colostomy--the Port Harcourt experience. West Afr J Med.

[18] Almosallam OI, Aseeri A, Shanafey SA (2016). Outcome of loop versus divided colostomy in the management of anorectal malformations. Ann Saudi Med.

[19] Adamou H, Habou O, Amadou Magagi I, Adakal O, Magagi A, Halidou M (2018). Pattern of Lower Intestinal Ostomies in a Low-Income Country: Case of Southeast of Niger Republic. World J Surg.

[20] Dode CO, Gbobo LI (2001). Childhood colostomy and its complications in Lagos. East Central Afri J Surg.

[21] Chirdan LB, Uba FA, Ameh EA, Mshelbwala PM (2008). Colostomy for high anorectal malformation: an evaluation of morbidity and mortality in a developing country. Pediatr Surg Int.

[22] van den Hondel D, Sloots C, Meeussen C, Wijnen R (2014). To split or not to split: colostomy complications for anorectal malformations or hirschsprung disease: a single center experience and a systematic review of the literature. Eur J Pediatr Surg.

[23] Oda O, Davies D, Colapinto K, Gerstle JT (2014). Loop versus divided colostomy for the management of anorectal malformations. J Pediatr Surg.

[24] Levitt MA, Pena A (2007). Anorectal malformations. Orphanet J Rare Dis.

[25] Youssef F, Arbash G, Puligandla PS, Baird RJ (2017). Loop versus divided colostomy for the management of anorectal malformations: a systematic review and meta-analysis. J Pediatr Surg.

